# Fibroblast mitochondria in idiopathic Parkinson’s disease display morphological changes and enhanced resistance to depolarization

**DOI:** 10.1038/s41598-020-58505-6

**Published:** 2020-01-31

**Authors:** P. M. A. Antony, O. Kondratyeva, K. Mommaerts, M. Ostaszewski, K. Sokolowska, A. S. Baumuratov, L. Longhino, J. F. Poulain, D. Grossmann, R. Balling, R. Krüger, N. J. Diederich

**Affiliations:** 1 LCSB, University of Luxembourg, Belvaux, Luxembourg; 2 IBBL, Luxembourg, Luxembourg; 3 CHL, Luxembourg, Luxembourg; 4 Transversal Translational Medicine, Luxembourg Institute of Health (LIH), Strassen, Luxembourg

**Keywords:** Cellular neuroscience, Parkinson's disease

## Abstract

Mitochondrial dysfunction is a hallmark in idiopathic Parkinson’s disease (IPD). Here, we established screenable phenotypes of mitochondrial morphology and function in primary fibroblasts derived from patients with IPD. Upper arm punch skin biopsy was performed in 41 patients with mid-stage IPD and 21 age-matched healthy controls. At the single-cell level, the basal mitochondrial membrane potential (Ψm) was higher in patients with IPD than in controls. Similarly, under carbonyl cyanide 4-(trifluoromethoxy)phenylhydrazone (FCCP) stress, the remaining Ψm was increased in patients with IPD. Analysis of mitochondrial morphometric parameters revealed significantly decreased mitochondrial connectivity in patients with IPD, with 9 of 14 morphometric mitochondrial parameters differing from those in controls. Significant morphometric mitochondrial changes included the node degree, mean volume, skeleton size, perimeter, form factor, node count, erosion body count, endpoints, and mitochondria count (all P-values < 0.05). These functional data reveal that resistance to depolarization was increased by treatment with the protonophore FCCP in patients with IPD, whereas morphometric data revealed decreased mitochondrial connectivity and increased mitochondrial fragmentation.

## Introduction

Multiple clues for mitochondrial dysfunction have been reported in idiopathic Parkinson’s disease (IPD), including defects in different complexes of the respiratory chain, altered mitochondrial membrane potential, increased oxidative stress, and decreased mitophagy^1^. However, evaluating the underlying mechanisms in living patients with IPD remains challenging.

Studies of mitochondrial dysfunction in easily accessible, non-neuronal tissues are needed to understand the mechanisms of dysregulation. Fibroblasts have been used to study the underlying pathophysiological mechanisms of IPD or genetic forms of Parkinsonism, as early reports revealed similarities in mitochondrial dysfunction between fibroblasts and neurons^2–11^. However, patients with IPD show no clinical signs of fibroblast pathology, suggesting that in this tissue type, compensatory mechanisms may overcome mitochondrial dysfunction.

In our previous studies of the tissue-dependent impact of mitochondrial dysfunction in IPD, we explored two different sample types in patients with mid-stage IPD: platelets and enteric submucosal ganglia. We observed no significant changes in mitochondrial function in IPD platelets, whereas the ganglia showed substantial morphometric changes in the mitochondria^12,13^. However, enteric submucosal ganglia must be obtained by deep colonic biopsy, preventing the use of this technique in large patient cohorts because of the invasiveness of the procedure and associated risks. Here, we examined fibroblasts, which are easily accessible using low-risk procedures and can be used for both in-depth morphological and functional analyses.

## Results

### Mitochondrial function

**Figure 1:**
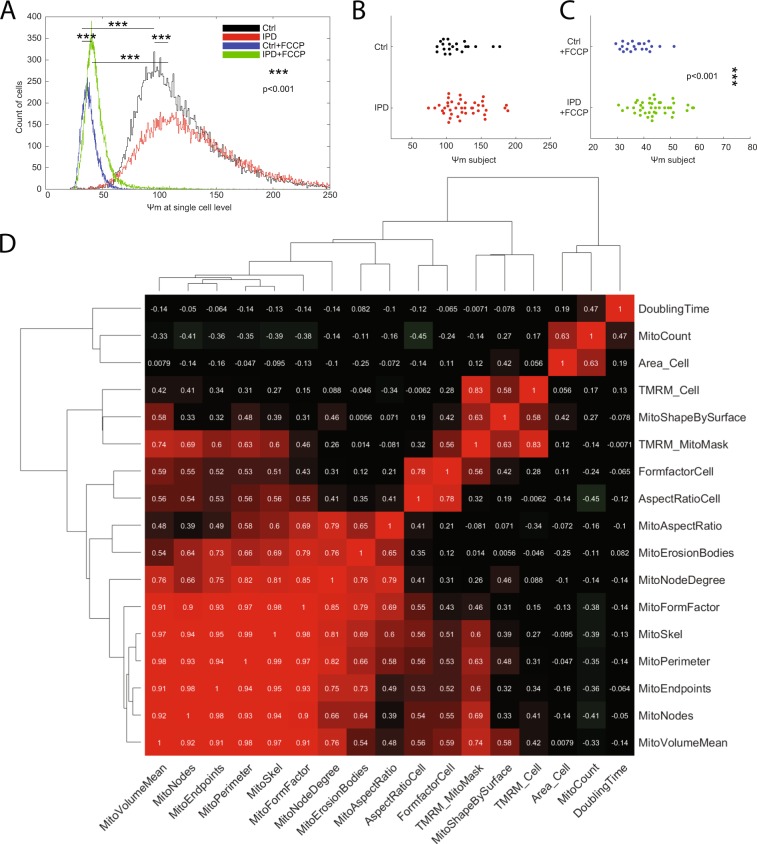
Increased mitochondrial membrane potential in fibroblasts derived from patients with IPD. (**A**) Analysis performed at baseline respectively after FCCP perturbation at the single-cell level. The histograms show the mean TMRM fluorescence in the cell area. Statistics: Wilcoxon rank-sum test, ***indicates P < 0.001. Counts of analysed cells per group: Ctrl unperturbed, 22.136; IPD unperturbed, 28.005; IPD + FCCP, 29.811; Ctrl + FCCP, 20.789. (**B**) Analysis performed at baseline at the level of individual subjects. Individual analysis at baseline condition showed no significant difference in TMRM fluorescence between patients and controls (P = 0.065). (**C**) Analysis performed after FCCP perturbation at the individual subject level. The difference in TMRM fluorescence between patients with IPD and controls was significant. Statistics: Wilcoxon rank-sum test, ***indicates P < 0.001. (**D**) Analysis of correlation between pairwise features. Pearson coefficients of correlation are provided as white text and color-coded. Dendrograms illustrate hierarchical clustering with Euclidean distance metric and average linkage. Correlations between cellular doubling time and cellular morphological parameters were negligible (correlation coefficient |r| < 0.30). Furthermore, mostly negligible (|r| < 0.30) and one low (0.30 < |r| < 0.50) but no moderate (0.50 < |r| < 0.70) or higher correlations were found between doubling time and functional or morphometric mitochondrial features.

Comparison of IPD cells and control cells revealed increased mitochondrial membrane potential in IPD fibroblasts under baseline conditions (Fig. 1A, P < 0.001). After mitochondrial depolarization with the protonophore carbonyl cyanide 4-(trifluoromethoxy)phenylhydrazone (FCCP), tetramethylrhodamine, methyl ester (TMRM) fluorescence remained significantly higher in patient-derived fibroblasts (Fig. 1A, P < 0.001). Comparison of the mean TMRM fluorescence for individual subjects confirmed a higher resistance to FCCP perturbation in patients with IPD than in controls, but no difference in the absence of FCCP (Fig. 1B,C, P = 0.065).

### Cell growth

**Figure 2:**
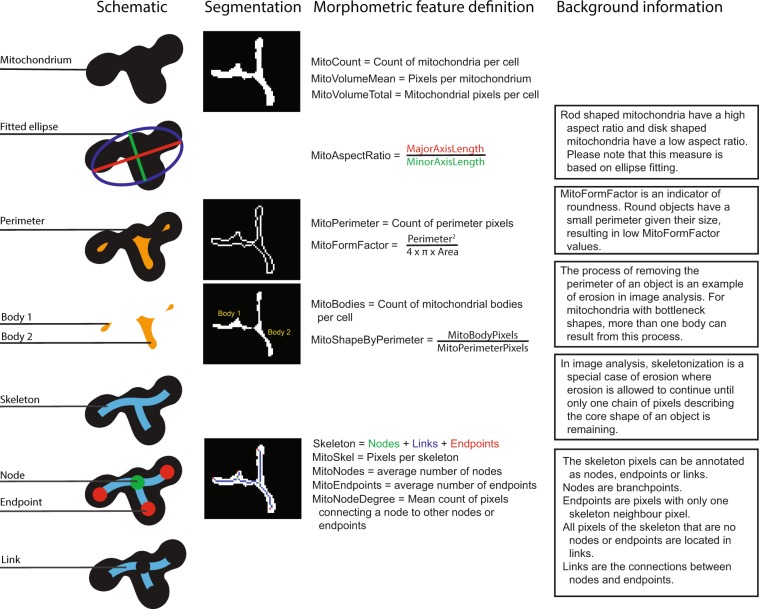
Morphometric features. The features are numeric descriptors of image contained information. The terminology used to define the features is illustrated in the schematic column. The segmentation column shows from top to bottom, a real example of a computationally defined mitochondrial region of interest (ROI), the perimeter of that ROI, the bodies that are remaining when removing the perimeter from the ROI, and an illustration of skeleton (blue, green, and red pixels), nodes (green pixels), and endpoints (red pixels). Please note that the terminology originates from computer vision and graph theory. Aspect ratio and form factor were used for both mitochondria and cells.

**Figure 3:**
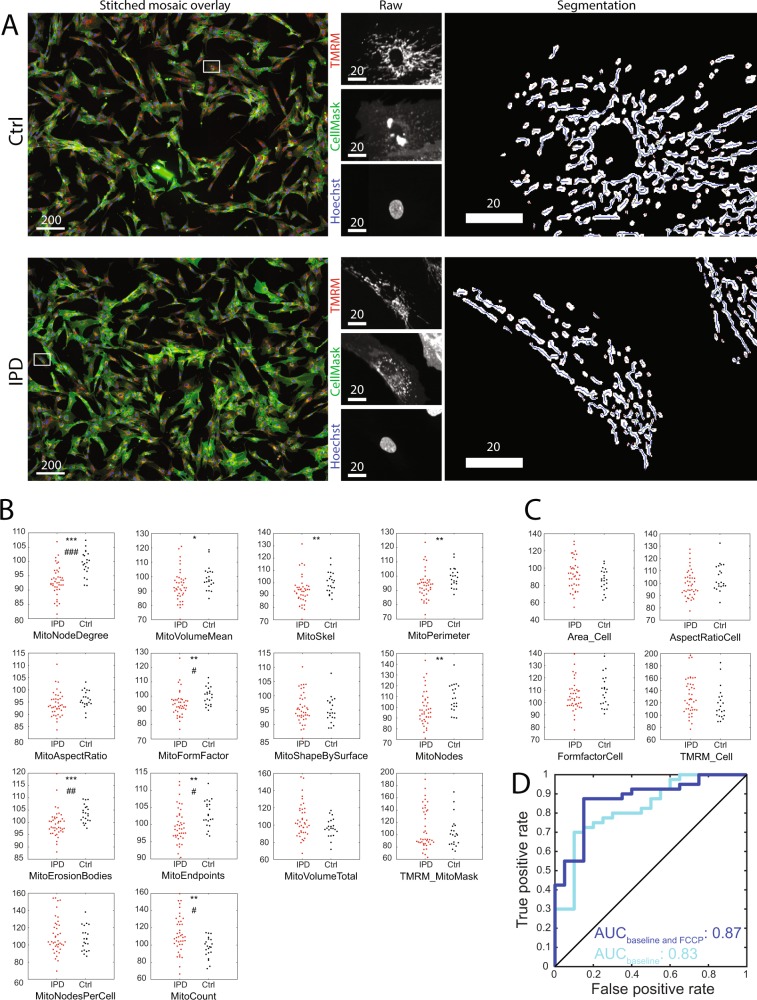
Cellular and mitochondrial morphometric characteristics in fibroblasts derived from patients with IPD and healthy controls as well as classification into the two groups based on these characteristics. (**A**) Segmentation examples. Please note the reduced branching of mitochondria in the IPD example compared to the Ctrl example. Scale bars: 20 or 200 µm, as labelled. White boxes in stitched mosaic overlays indicate coordinates of raw image examples. (**B**) Individual subject analysis of mitochondrial morphometric features. The mean TMRM intensity within mitochondria at the base level is shown as TMRM_MitoMask. (**C**) Individual subject analysis of cellular morphometric features. The mean TMRM intensity within cells at the base level is shown as TMRM_Cell. Statistics and significance: without Bonferroni correction: [*P < 0.05; **P < 0.01; ***P < 0.001]; with Bonferroni correction: [^#^P < 0.05; ^##^P < 0.01; ^###^P < 0.001]. The minimum number of cells analysed per individual: 175. (**D**) Classification into patients with IPD and healthy controls by morphofunctional characteristics. A random classification would result in a diagonal line (shown in black) in the receiver operating characteristic (ROC) and an area under receiver operating characteristic curve (AUC) of 0.5, whereas a perfect classification would result in AUC = 1. This ROC shows that predictions learned from pooled data that take into account mitochondrial and cellular morphometrics including both baseline and FCCP conditions, provide a better predictive power than baseline data alone (baseline alone, cyan, AUC = 0.83; baseline and FCCP, dark blue, AUC = 0.87).

During the expansion of primary fibroblasts, the doubling time was significantly increased in IPD samples compared to control samples (Table 1, P < 0.001). We evaluated whether the doubling time was correlated with mitochondrial function and morphometrics and found that the correlations were negligible, except for a low correlation with the mitochondria count (Fig. 1D, r = 0.47). The negligible correlations between the doubling time and cellular form factor or cellular aspect ratio exclude a changed cell type composition of fibroblasts and morphologically different keratinocytes in IPD. Furthermore, despite the lower growth rate of cells from IPD samples, the cellular morphometric features (Fig. 2) aspect ratio, and form factor, and total mitochondrial volume did not differ between IPD and control fibroblasts (Fig. 3, MitoVolumeTotal P > 0.05, AspectRatioCell P > 0.05, FormFactorCell P > 0.05).

**Table 1:** Demographics and cellular doubling time (mean +− standard deviation).

	IPD	Controls	P-value
Age (mean +− SD) [years]	66.3 ± 7.3	66.1 ± 5	0.901
Gender M/F	25/16	4/17	0.001
PD duration	6.6 ± 5.2	NA	NA
UPDRS motor score	10.4 ± 5	0.6 ± 1	<0.001
Levodopa dosage	589.8 ± 348.9	NA	NA
MMSE score	27.9 ± 4.7	29.1 ± 1.3	0.172
FAB score	15.4 ± 2.5	16 ± 2.6	0.197
UPSIT score	19.5 ± 7.1	31 ± 4.7	<0.001
NMS score	8.1 ± 5.3	2.7 ± 2.5	<0.001
Doubling time [days]	16.1 ± 18.5	4.9 ± 4.2	<0.001

Abbreviations: UPDRS = Unified Parkinson’s Disease Rating Scale; MMSE = MiniMental State Examination; FAB = Frontal Assessment Battery; UPSIT = University of Pennsylvania Smell Identification Test; NMS = Non-motor symptom score.

### Multiple linear regressions

The influence of mitochondrial morphology on TMRM fluorescence, independently from disease status, was analysed by multiple linear regression. The linear model revealed a significant negative influence of mitochondrial branching (MitoSkel, P < 0.001, *t* value −8.9; MitoNodeDegree, P < 0.01, *t* value −2.9) and positive influences of mitochondrial volume (MitoVolumeMean, P < 0.001, *t* value 13.2) and mitochondrial endpoints (P < 0.001, *t* value 6.4) on TMRM fluorescence in the absence of FCCP (Table 2). Thus, less-branched mitochondria showed higher membrane potentials.

**Table 2:** Mitochondrial TMRM fluorescence correlates with mitochondrial morphometric features.

	Estimate	t value	P-value
MitoVolumeMean	7.5180	13.246	<2e−16
MitoSkel	−6.7891	−8.911	2.51e−12
MitoEndpoints	5.1302	6.363	3.89e−08
MitoNodeDegree	−1.4292	−2.899	0.00533
PD diagnosis	7.8065	2.255	0.02806

Multiple linear regression summary with multiple R-squared = 0.8857.

### Morphometrics

We found mitochondrial morphometric changes between patients with IPD and age-matched controls in 9 of 14 features (Fig. 3B); the increased mitochondrial count (MitoCount, P < 0.01), decreased mean mitochondrial size (MitoVolumeMean, P < 0.05), and unchanged total mitochondrial mass per cell (MitoVolumeTotal, P > 0.05) revealed mitochondrial fragmentation in IPD. Thus, fibroblasts from patients with IPD contained more mitochondria than those from healthy controls, but the total mass of mitochondria (MitoVolumeTotal) was not increased because the fragmented mitochondria reduced the average mitochondrial mass (MitoVolumeMean). Notably, features associated with the mitochondrial perimeter (MitoPerimeter P < 0.01, MitoFormFactor P < 0.01, MitoErosionBodies P < 0.001) and mitochondrial branching (MitoNodeDegree P < 0.001, MitoSkel P < 0.01, MitoNodes P < 0.01, MitoEndpoints P < 0.01) were changed in IPD. In contrast, the parameter based on ellipse fitting (MitoAspectRatio P > 0.05) did not differ. Overall, the data showed reduced mitochondrial branching in IPD fibroblasts. Because of the gender imbalance in the study cohort (Table 1), we evaluated the influence of gender on the results. To correct for the eventual influence of age or gender, we included both variables as covariates in the linear model (Table 2). Age and gender were not significant covariates affecting TMRM fluorescence, and there was no significant difference in mitochondrial branching between male and female subjects.

### Classification

Based on the morphometric and functional mitochondrial data, the study participants were classified into the IPD and control groups by using support vector machines. To evaluate the predictive power of this classification method, the area under the receiver operating characteristic curve (AUC) was measured (Fig. 3D). When only using baseline mitochondrial and cellular data, the AUC was 0.83. The combination of baseline and FCCP challenge data improved the classification performance to AUC = 0.87.

## Discussion

Although fibroblasts display a lower bioenergetic burden than neurons, mitochondrial changes such as reduced pyruvate oxidation and inhibition of the activities of complexes I, IV, and V have been reported in IPD-derived fibroblasts, confirming that energy metabolism is decreased in these cells^4,11,14,15^. In this study, we observed substantial morphometric and functional changes of fibroblasts from patients with IPD compared to age-matched healthy controls. We combined functional and in-depth morphometric analyses in the same cellular screening assay as an innovative approach. Moreover, fibroblasts are easily accessible, and the image analysis and machine learning approaches provide a screenable phenotype allowing for automated classification of a healthy or IPD status from skin biopsies as well as identification of biomarkers. As these skin punch biopsies are not invasive and easily performable in outpatients, and the morphological and functional mitochondrial analysis method established here is largely automated with interpretation performed by a machine learning method, this technique can be used to screen patients with IPD. Beyond the clinical description of patients, objective cellular data may be useful as disease progression markers when screening for disease-modifying compounds.

At the stage of sample preparation, IPD fibroblasts showed an increased doubling time, contrasting the results of previous studies, although increased mitochondrial membrane potential and actively respiring mitochondria were observed in both IPD and control fibroblasts in another study^8^. The increased doubling time in IPD may be related to senescence or changes in the abundance of keratinocytes in primary fibroblast cultures. We have excluded the second option, as the correlation between the cellular doubling time and cellular morphology was negligible. Thus, fibroblasts derived from patients with IPD may have a higher susceptibility for cellular senescence^16^.

Our study provides highly detailed information on the mitochondrial morphology, suggesting reciprocal compensatory interaction between the mitochondrial membrane potential and mitochondrial morphology. We used the TMRM subquenching method in combination with an FCCP challenge test for the relative quantification of mitochondrial membrane potential^17^. Increased resistance to FCCP-induced depolarization was significant at the single-cell level and in individual subject analysis. In contrast, increased TMRM fluorescence at baseline was only significant at the level of statistically better powered single-cell analysis: in each of the 41 patients and 21 healthy controls, at least 175 cells were analysed. The increased resistance to mitochondrial depolarization agrees with increased levels of reactive oxygen species and a proton leak in IPD, as reported previously^8^. Indeed, the proton leak reported by others in IPD fibroblasts^8^ was indirectly quantified as a compensatory increase in the oxygen consumption rate. We observed hyperpolarization of mitochondrial membranes by single-cell analysis, suggesting overcompensation for mitochondrial stress in IPD fibroblasts. Furthermore, the correlation between mitochondrial morphometrics and membrane potential suggests a link between the shape and function of mitochondria. However, our findings contrast those found in familial forms of parkinsonism; particularly, LRRK2 G2019S^18^ and several Parkin mutants^5^ displayed decreased mitochondrial membrane potential.

Our data will enable further research of Parkinson’s disease subtype stratification and shall motivate further research on the underlying mechanisms, including the activities of mitochondrial complexes and metabolic pathways. Mitochondrial dysfunction has previously been observed in both fibroblasts and other cell models, such as induced pluripotent stem cell-derived neurons derived from patients with IPD^4,11,14,15^. The cell-type-specific vulnerability of cellular metabolism specific for a predefined cellular phenotype has been proposed in PINK1-associated Parkinson’s disease^19^. The present study furthermore quantified and revealed significant mitochondrial morphometric changes previously reported in IPD fibroblasts^8^. Increased numbers of mitochondria and reduced average mitochondrial volume and branching provide quantitative and technically reproducible evidence for mitochondrial fragmentation. This fragmentation agrees with the upregulation of autophagy and observed mitophagy events in IPD fibroblasts^8^. A key pathway in the regulation of mitophagy involves the familial Parkinson’s disease-associated proteins PINK1 and Parkin and outer mitochondrial membrane protein Miro^20^. A recent study showed that in IPD fibroblasts, degradation of Miro and subsequent mitochondrial clearance were impaired^21^. In combination with increased mitochondrial numbers and previously observed ongoing mitophagy events, these findings suggest that PINK1-mediated initiation of mitochondrial degradation is impaired in IPD fibroblasts^8^.

The increased number of mitochondria agrees with previous findings for enteric ganglia from the left colon in patients with IPD^13^. In fibroblasts, we found that an increased number of mitochondria was not associated with an increase in the total mitochondrial mass, suggesting that enhanced resistance to FCCP-mediated depolarization is associated with an increased surface to volume ratio and potentially associated with an increased density of mitochondrial complexes.

Notably, different familial forms of PD manifest different functional and morphometric mitochondrial traits, demonstrating that patient stratification based on mitochondrial characteristics may lead to PD-subtype targeted disease-modifying therapies^1,5,10^.

The presence of functional and morphometric mitochondrial changes in IPD fibroblasts agrees with the suggestion that cellular hallmarks associated with IPD involve impairments in highly conserved pathways^22–24^. While energetic stress and vulnerability are eminent in dopaminergic neurons, defects in conserved pathways affect a multitude of cell types beyond dopaminergic neurons, which may be measurable at a cellular level even if they do not emerge in higher tissues, in organs, or at clinical levels^25,26^. Indeed, these cellular hallmarks may exist ubiquitously but cell death may only emerge in the most vulnerable neurons, such as pace-making dopaminergic neurons in the *substantia nigra* undergoing sustained energetic stress^27,28^. As patients with IPD have no clinical symptoms because their fibroblasts are impaired, the mitochondrial adaptations in these cells may be at least partially successful^29^. Although no direct connections have been identified between fibroblasts and the clinical symptoms of IPD, the mitochondrial phenotypes observed in fibroblasts provide disease-specific mitochondrial morphofunctional data that can, at least in part, be extrapolated to energetically more active and vulnerable cells such as neurons. Thus, it is reasonable that differences in mitochondrial membrane potential between subjects with or without IPD were only found under conditions of FCCP challenge.

As computer vision concepts, the morphometric features have no direct functional consequences in the biological sense. Nevertheless, they describe morphology in a quantitative and reproducible manner, and mitochondrial morphology and function have been shown to be associated^8,30,31^. Mitochondrial fusion can prevent increases in the local concentration of defective mitochondrial proteins by dilution; reciprocally, accumulation of damaged macromolecules can be eliminated within mitochondrial units by separation from the mitochondrial network^32^. Despite considerable progress in the understanding of mitochondrial morphofunction in the last decade, the bidirectional links between mitochondrial external shape, internal structure, and function have not been comprehensively and quantitatively determined^33^. Our results may enable further progress by providing quantitative mitochondrial morphometric data in an automated high-throughput manner.

Our study has numerous strengths. In terms of the number of participants, this was the largest study of mitochondrial dysfunction in IPD primary fibroblasts, the quantitative computational image analysis showed reproducible results, and all fibroblasts were analysed at the same low passage number^1,2^. We extended established standards in the mitochondrial morphometric analysis to capture the detailed properties of the mitochondrial perimeter and skeleton. The sensitivity of our study was higher than that of existing approaches which use approximations such as ellipse fitting. Indeed, aspect ratio analysis involves mathematical fitting of an ellipse and then divides the length of the ellipse’s major axis by its minor axis length^34^. For non-branched mitochondria, the fitting of an ellipse is reasonable. However, more complex shapes cannot be appropriately described with an ellipse. Graph theory features, in contrast to analysis of the aspect ratio, do not involve mathematical fitting of an ellipse to a mitochondrion but rather skeletonization. Because skeletonization maintains the mitochondrial topology, it can discriminate phenotypes that are indistinguishable in aspect ratio analysis^35^. In combination with machine learning, the identified IPD phenotype and low invasiveness of the procedure for patients make this novel approach applicable as a screening assay in future large-scale studies.

There were some limitations to this study. Although there were no clinical signs of genetic Parkinsonism, formal genetic testing was not performed, as these tests were not included in the consent form. However, fibroblasts derived from patients with Parkinson’s disease with the LRRK2 mutation, which are clinically indistinguishable from those of IPD in terms of disease onset and main motor symptoms, showed a different set of mitochondrial morphometric changes than in patients with IPD analysed in the present study, particularly mitochondrial elongation and increased interconnectivity^18^. In contrast, other cell types carrying the LRRK2 G2019S mutation contain large numbers of mitochondria and show increased mitochondrial fragmentation but reduced mitophagy^36,37^. The gender imbalance in the present study was because of the low proportion of male controls and may have masked potential gender-specific phenotypes. However, our analysis indicated that age and gender did not significantly impact the measured mitochondrial morphology. The resolution limit of microscopy affects the samples derived from patients with Parkinson’s disease and controls; consequently, the significant differences are valid, but a lack of significance may be due to the resolution limit. Finally, mitochondrial changes are extremely dynamic, and not fully captured by cell population snapshots analyzed at the single-cell level.

In summary, we established a screenable assay for mitochondrial function and morphology in primary fibroblasts. This assay leverages extensive cohort fibroblast screening and phenotype-based sample stratification, leading to better stratification of Parkinson’s disease subtypes, which may lead to endophenotype-specific and subtype-specific therapeutic strategies.

## Conclusions

We segmented IPD and control group-derived fibroblasts based on mitochondrial morphometrics and mitochondrial membrane potential under FCCP stress. Mitochondrial fragmentation was associated with increased resistance to FCCP induced depolarization in patients with IPD. Our results contribute to the robust evidence for peripheral non-neuronal cellular hallmarks in IPD, as primary fibroblasts showed functional changes in their mitochondria under FCCP stress as well as morphometric changes in their mitochondria, which were detectable even in the absence of such mitochondrial stress. In-depth analysis of fibroblast mitochondria can be used for translational research, not only in genetic forms of Parkinson’s disease but also in IPD.

Our method should be used to study fibroblasts in replication cohorts with much larger numbers of patients to evaluate mitochondrial morphology and function. Indeed, the predictive power qualifies the developed assay for future large-scale studies requiring screenable phenotypes.

## Methods

### Subjects

The Luxembourg “Comité National d´Éthique de la Recherche (CNER)” approved the study (decision 201107/03). Informed written consent was obtained from all study participants (Table 1). All methods were carried out following relevant guidelines and regulations.

Forty-one non-demented patients with IPD were prospectively recruited through the Neurological Clinic of the *Centre Hospitalier de Luxembourg*. The 21 healthy control subjects were unrelated family members and support group members. The general clinical status of all subjects was evaluated. No subjects had dermatologic or rheumatologic disease. The IPD stage was rated based on the Hoehn-Yahr scale and UPDRS motor scale while the patients were being administered their usual medications.

### Preparation of primary fibroblasts

After local anaesthesia with 2% lidocaine, 3-mL standardized punch skin biopsies were obtained at the lateral part of the upper arm in all subjects by the same dermatologist. Skin biopsies were transported in sterile tubes containing transport media [DMEM + GluMAX-I (21885-025, Gibco, Grand Island, NY, USA) + 1% Pen/Strep (Gibco, 15140-122)]. Skin biopsies were cultured within 4 h of collection and allowed to adhere before expansion under standardized conditions in culture media [DMEM + GluMAX-I (Gibco, 21885-025) + 10% foetal bovine serum (FBS; Gibco, 16140-063) + 1% Pen/Strep], in 5% CO_2_ at 37 °C. Each passage was performed using Trypsin/EDTA solution (CC-5012, Lonza, Basel, Switzerland) when the fibroblasts reached 70–90% confluence. Fibroblasts from the first passage were cryopreserved in cryoprotectant [DMEM + GluMAX-I + 20% FBS + 10% dimethyl sulfoxide (D2650, Sigma, St. Louis, MO, USA)] in a controlled-rate freezer with the temperature lowered from 4 °C to −80 °C at 1 °C/min. The cryopreserved cells were kept in liquid nitrogen vapor. The concentration of total fibroblasts cryopreserved was 1–2 × 10^6^ cells in 1 mL of cryoprotectant. The absence of mycoplasma contamination was verified using MycoAlert^TM^Plus (Lonza, LT07‐703). To assure sample backups and enough sample for high-content screening for each study participant, one cryovial from the first passage was thawed, and the cells were cultured as described above until third passage cryopreservation. One cryovial of the third passage was used per subject for shipment to the analysis laboratory. The doubling time was defined between passage two and passage three, as previously reported with increased doubling time indicating slower growth^8^. Thawed cells from passage three were expanded and passaged for transfer into assay plates as described below. All assays were performed using cells from passage 4.

### Cell culture

Freezing stocks were thawed in a 37 °C water bath and transferred into 5 mL prewarmed growth medium (DMEM + GluMAX-I + 10% FBS + 1% Pen/Strep) in 25-cm2 flasks. The media were changed 24 h after seeding and then every 48 h until the culture reached 80–100% confluency.

### Staining and challenge test

A total of 1 × 10^4^ cells per well was seeded into CellCarrier 96-well plates (6005550, PerkinElmer, Waltham, MA, USA) and incubated for 24 h before staining. For staining with tetramethylrhodamine methyl ester (TMRM, T668, Invitrogen, Carlsbad, CA, USA), Hoechst 34580 (Invitrogen, H21486), and CellMask Deep Red plasma membrane stain (C10046, Life Technologies, Carlsbad, CA, USA), a master staining mix in DMEM + GluMAX-I + 10% FBS + 1% Pen/Strep containing 10 nM TMRM, 1 µg/mL Hoechst, and 0.5x CellMask was prepared. Next, 200 µL of staining master mix was added to each well and the samples were incubated in the dark at 37 °C for 30 min. To remove the CellMask and Hoechst without changing the TMRM conditions, the staining mix was replaced with growth medium containing 10 nM TMRM and optionally 40 µM carbonyl cyanide 4-(trifluoromethoxy)phenylhydrazone (FCCP, C2920, Sigma) as shown in the results. Before acquiring images, the samples were incubated at 37 °C for 20 min.

Images were acquired on an Opera quadruple excitation high sensitivity spinning disk microscope with incubation conditions set to 37 °C, 80% humidity, and 5% CO_2_. In the first exposure, TMRM was excited with a 561-nm laser and detected behind a 600/40 bandpass filter. In the second exposure, Hoechst and CellMask deep red were measured simultaneously. Hoechst was excited with a 405-nm laser and detected behind a 450/50 bandpass filter. CellMask was excited with a 640-nm laser and detected behind a 690/70 bandpass filter. To fit large cell parts on single tiles and ensure sufficient resolution for mitochondrial analysis, a 20x water immersion objective (numerical aperture = 0.7) was used. One 5 × 5 mosaic was acquired in each well. The experimentalist was blinded to the sample status during all procedures.

### Image analysis

Image analysis was performed using Matlab 2016b (Mathworks, Natick, MA, USA) and established computer vision concepts^38^ (Fig. 2). Mitochondrial parameters illustrated in Fig. 2 were defined with randomly selected images from the present study, with the programmer blinded for the sample status. In the following text, Matlab function calls are highlighted in courier new font in the format function Name(argument).

For nuclei detection, the Hoechst channel was low-pass-filtered with a Gaussian filter of size 10 and standard deviation 2 and thresholded (>100). Objects with fewer than 500 pixels were removed from the nuclei mask. The cell area was defined using the difference in Gaussians and thresholding on the CellMask deep red channel. Objects with fewer than 200 pixels were removed.

To split single cells, we implemented an algorithm combining information from all three channels. Splitting was implemented using grey tone erosion, as organelle membranes around the nucleus enable estimation of the cell shape. Briefly, the cell splitting algorithm evaluates the number of nuclei per connected component and iteratively splits via grey tone erosion. Grey tone erosion was implemented using the function imerode(disk-shaped structuring element, radius 12). Splitting success was tested by nucleus counting.

Mitochondria were segmented using the difference of Gaussians and subsequent thresholding. Objects with fewer than 6 pixels were removed from analysis (MitoMask). Mitochondrial skeletonization was computed using a published algorithm based on the homotopic thinning algorithm^39,40^. Nodes and endpoints were computed using Matlab’s bwmorph function. MitoBodies were defined via erosion of the mitochondrial mask, using imerode(disk-shaped structuring element, radius 1). The mitochondrial perimeter was defined by subtracting MitoBodies from MitoMask. The mitochondrial features described in Fig. 2 were computed for each mitochondrion to compute the mean values per cell for downstream analysis.

### Statistics

The following statistical tests were applied. For data with a normal distribution, analysis was performed by unpaired *t*-test with Welch’s correction. For data showing a symmetric distribution, Mann-Whitney U-test was used. For data with a non-symmetric distribution, a permutation test with 10^7^ permutations was used. Categorical values were analysed by Fisher’s exact test. Notably, in cases of multiple testing, the resulting P-values were adjusted by Bonferroni correction. Symmetry was analysed using two published methods, implemented in the lawstat R package^41–43^. The distribution of data was considered as asymmetric if any of the tests indicated asymmetry.

Correlations between pairwise features, including doubling time, cellular morphology, mitochondrial morphology, and TMRM fluorescence were calculated using Pearson’s linear correlation coefficient (r). Correlations with |r| < 0.30 were considered as negligible, correlations with 0.30 < |r| < 0.50 were considered as low, and correlations with 0.50 < |r| < 0.70 were considered as moderate^44^.

The impact of mitochondrial morphometrics and clinical variables on the mitochondrial membrane potential was assessed by multiple linear regression implemented using the lm function in R programming language.

Classification using support vector machines, cross-validation procedures, and receiver operating characteristic were implemented as previously reported^13^.

## Data Availability

Anonymized data will be provided upon request and under the umbrella of the European data protection legislation.
